# The last known freshwater coelacanths: New Late Cretaceous mawsoniid remains (Osteichthyes: Actinistia) from Southern France

**DOI:** 10.1371/journal.pone.0234183

**Published:** 2020-06-05

**Authors:** Lionel Cavin, Eric Buffetaut, Yves Dutour, Géraldine Garcia, Jean Le Loeuff, Annie Méchin, Patrick Méchin, Haiyan Tong, Thierry Tortosa, Eric Turini, Xavier Valentin

**Affiliations:** 1 Department of Geology and Palaeontology, Natural History Muesum, Geneva, Switzerland; 2 Laboratoire de Géologie de l’Ecole Normale Supérieure, CNRS (UMR 8538), PSL Research University, Paris, France; 3 Palaeontological Research and Education Centre, Mahasarakham University, Kantarawichai, Maha Sarakham, Thailand; 4 Aix-en-Provence, Muséum d’Histoire Naturelle, Paris, France; 5 Laboratoire de Paléontologie, Evolution, Paléoécosystèmes et Paléoprimatologie, UMR CNRS 7262 Université de Poitiers, Poitiers, France; 6 Musée des Dinosaures, Espéraza, France; 7 Musée de Cruzy, Cruzy, France; 8 Département des Bouches-du-Rhône, Hôtel du Département, Réserve Naturelle de Sainte-Victoire, Marseille, France; 9 Palaios, Morthemer Valdivienne, France; Università degli Studi di Torino, ITALY

## Abstract

Coelacanths are iconic fishes represented today by a single marine genus. The group was a little bit more diversified in the Mesozoic, with representatives in marine and continental environments in the Late Cretaceous. Here we describe isolated skull bones of the last know freshwater coelacanths found in several fossil sites from the Early Campanian to the Early Maastrichtian of Southern France (in the Departments of Aude, Bouches-du-Rhône, Hérault, and Var). The sample does not allow distinguishing different species, and all material is referred to *Axelrodichthys megadromos* Cavin, Valentin, Garcia originally described from the locality of Ventabren in Southern France. A reconstruction of the skull is proposed. Previously unrecognized features are described, including parts of the postparietal portion of the skull, of the suspensorium and of the mandible. The new data confirm the assignation of the species to the mawsoniids, and more specifically to *Axelrodichthys*. A cladistic analysis scoring new character states provides a similar topology than a previous analysis, i.e. *A*. *megadromos* is placed in a polytomy with *Axelrodichthys araripensis* and *Lualabaea lerichei*, two species from the Early Cretaceous of Brazil and from the Late Jurassic of the Democratic Republic of the Congo, respectively. *A*. *megadromos* appears to have been restricted to freshwater environments, to the contrary of oldest Western Gondwanan representatives of the family that were able to live in brackish and marine waters. *A*. *megadromos* is the last representative of the mawsoniids and its occurrence in Europe is probably the result of a dispersal event from Western Gondwana that happened somewhen in the Cretaceous. Based on the available data, the mawsoniids went extinct in the mid-Maastrichthian, i.e. before the end-Cretaceous mass extinction. But it is possible that the fossil record of this family, which has been only recently recognized in Late Cretaceous European deposits, will geographically and stratigraphically widen with further discoveries.

## Introduction

Coelacanths, or Actinistia, are known from the Early Devonian up to the present, with only two living species, *Latimeria chalumnae* and *L*. *menadoensis*. The clade was never specious, and it diversified a little in the Devonian and Carboniferous and attained a maximum of diversity in the Early Triassic [1]. During the Cretaceous coelacanths are known by two families only, the Latimeriidae, which were represented by a few genera and which survived to the present with the genus *Latimeria*, and the Mawsoniidae, which went extinct a few million years before the Cretaceous–Palaeogene boundary based on our current knowledge. In the Cretaceous, latimeriids were exclusively marine while mawsoniids were euryhaline, with species dwelling in fresh and brackish waters. For a long time, the Cretaceous fossil record of mawsoniids were restricted to Western Gondwana, i.e. South America and Africa. In 2004, an single mawsoniid remain was found in the Late Cretaceous of Madagascar [2] and one year later, a single lower jaw bone from the terminal Cretaceous of southern France was referred to a mawsoniid coelacanth [3], thus extending the geographical and stratigraphical ranges of the family. Recently, more complete cranial material belonging to a single specimen discovered in the Early Campanian of Ventabren (Bouches-du-Rhône Department, France) allowed to identify a new species of *Axelrodichthys*, *A*. *megadromos* Cavin, Valentin, Garcia 2016. *Axelrodichthys* is a genus previously known by two species from the early Cretaceous of Brazil, *A*. *araripensis* Maisey, 1986 and *A*. *maiseyi* Carvalho, Gallo, Santos, 2013 (see [4] for a discussion of the status of the latter species), but some evidence indicate that this genus, as currently defined, is also present in Africa [2], [4], [5], [6]. A recent phylogenetic analysis of the family [7] found a polytomy grouping the Cretaceous genera *Lualabaea*, *Axelrodichthys*, *Mawsonia* and, more surprising, the Jurassic marine genus *Trachymetopon*.

Here we describe new material from several Late Cretaceous (Campanian and Maastrichtian) sites of Southern France (Aude, Bouches-du-Rhône, Hérault and Var Departments). The new fossils show no significant differences with the type material of *A*. *megadromos*. Consequently, all the material known from the Campanian and Maastrichtian of Southern France is referred to this species, and a cranial reconstruction is proposed. The phylogenetic affinities and the ecology of this coelacanth are discussed.

## Localities and geological setting (Fig 1)

**Fig 1:**
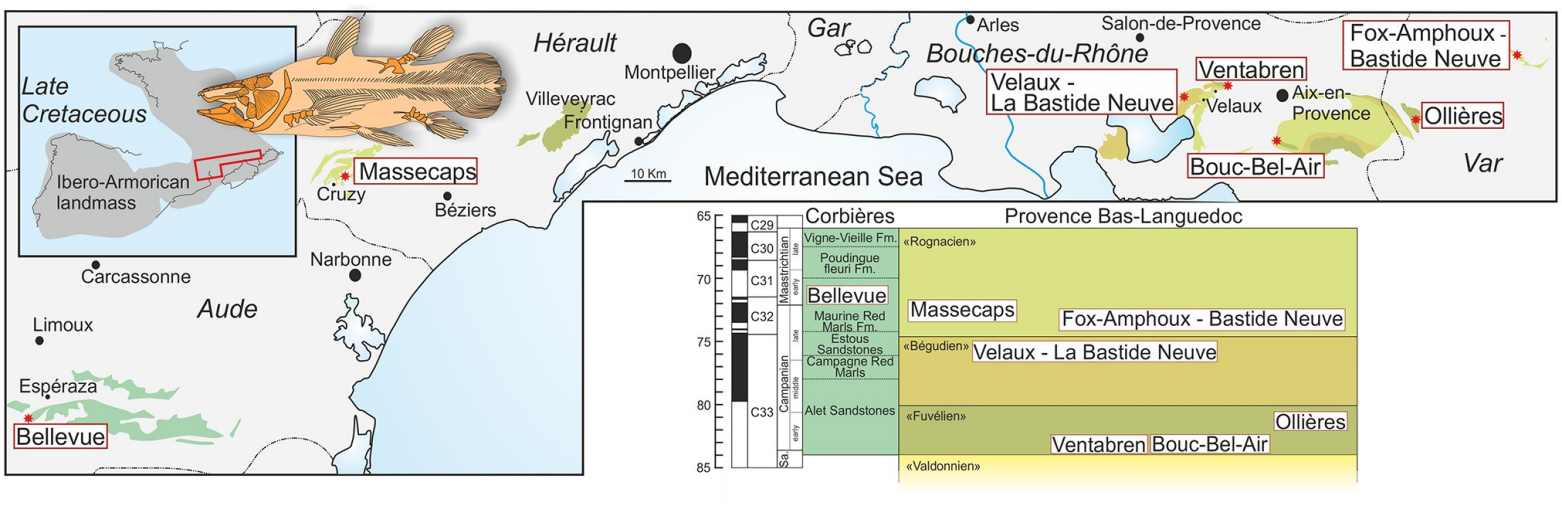
Map of Southern France showing the Campanian—Maastrichthian main outcrops with the localities that have yielded mawsoniid fossil remains. Geological information modified from http://infoterre.brgm.fr. The upper-left box shows the palaeogeographical reconstruction of the area in the Late Cretaceous (modified from Fondevilla et al. 2019 [40]). Stratigraphical chart showing correlations between lithostratigraphical units and the stratigraphical positions of the localities with mawsoniids remains (data from Laurent et al. 2001 [39], Tortosa 2014 [13], Fondevilla et al. 2019 [40]).

### Early Campanian (“Valdo-Fuvélien” facies) localities

#### Ventabren—Aire de Repos (Bouches-du-Rhône) (acronym: VAR)

This locality was discovered and excavated by the staff from the University of Poitiers. It has yielded a partial skull found by XV of a mawsoniid forming the type material of *Axelrodichthys megadromos* [8]. Other fish remains include bones of a gar (lepisosteid) referred to *Atractosteus africanus* [9] that was latter regarded as an indeterminate lepisosteid by Grande & Bemis [10]. Other vertebrates include crocodiles, pleurodiran and cryptodiran turtles. Works by the staff from the University of Poitiers with complementary researches by the staff from the Département des Bouches-du-Rhône and from the natural history museum of Aix (MHNAix) located the site at the limit between the “Valdonnien” and “Fuvélien” facies on the basis of a bivalve and gastropod assemblage (*Corbicula concinna*, *Campylostylus galloprovincialis*, *Viviparus bosqui* var. *novemcostata* and *Hadraxon acicula*) [11], [12]. This stratigraphic location corresponds to a Lower Campanian age.

#### Bouc-Bel-Air—Sousquières (Bouches-du-Rhône) (BAS)

The fossiliferous level was discovered by the staff from the University of Poitiers in collaboration with the natural history museum of Aix-en-Provence (MHNAix). Salvage excavations were conducted in 2018 by the staff of the Département des Bouches-du-Rhône and of the MNHAix. The lithology in Bouc-Bel-Air is rather similar to that of the Ventabren locality. It consists of beige and blackish marls and a brown lacustrine limestone layer, with the fossils located in a blackish marl layer. The fossil assemblage comprises gastropods, actinopterygian and mawsoniid fishes (this work), turtles, a large varanoid lizard, crocodiles and dinosaurs (dromaeosaurids). As in Ventabren, the lithology and the same molluscan assemblage completed by *Melania praelonga* and *Unio toulouzani* indicate a position close to the limit between the “Valdonnien” and “Fuvélien” facies, which corresponds to a Lower Campanian age [11], [12].

#### Ollières—Autoroute Nord/Sud sites (Var) (OLA)

The locality was discovered along the highway A8 [13]. It has yielded lepisosteids, amiid and mawsoniid fishes (this work), anuran and albanerpetontid amphibians, pleurodiran and cryptodiran turtles, squamates, crocodiles, and dinosaurs (nodosaurids and dromaeosaurids) [13].The locality is poorly dated. Based on the general litho- and biofacies, Ollières–Autoroute Nord/Sud corresponds to the «Fuvélien». It is however difficult to locate more precisely the site because of a lack of stratigraphical biomarkers. The locality is situated about 130 meters below the “Fuvélien”—“Bégudien” boundary, the fossiliferous bed is probably Early Campanian in age [13]. On the basis of the “Fuvélien” facies, the palaeoenvironement is interpreted as clearly lacustrine.

### Late Campanian (“Bégudien” facies) locality

#### Velaux—La Bastide Neuve (Bouches-du-Rhône) (VBN)

This site is excavated since 2002 by the team of the University of Poitiers and the Association Palaios. It has yielded the oldest-known freshwater crab [14] an abundant and diversified vertebrate fauna, including hybodont shark, and mawsoniid fish (this work), pleurodiran and cryptodiran turtles, the basal eusuchian crocodile *Allodaposuchus precedens* [15], the pterosaur *Mistralazhdarcho maggii* [16] and dinosaurs (the titanosaur *Atsinganosaurus velauciensis* [17], [18], the iguanodontian *Matherodon provincialis* [19], nodosaurid and abelisaurid [20]). The Late Campanian dating of the site is based on Magnetostratigraphic analyses together with biostratigraphic data from dinosaur eggshell and charophytes [21]. The palaeoenvironment is interpreted, on the basis of lithologic and taphonomic studies in association with microfacies and palynofacies, as a freshwater environment [14] corresponding to fluvial setting of moderate energy with broad floodplain.

### Late Campanian—Early Maastrichtian (“Rognacien” and Maurine Red Marls) localities

#### Cruzy—Massecaps and Montplo-Nord (Hérault) (CMA)

Excavations have been conducted during more than 20 years by the Association ACAP and staff from the CNRS. The composite vertebrate assemblage of these close localities comprises albanerpetontid and anuran amphibians, pleurodiran and cryptodiran turtles, a freshwater mosasaur, an azhdarchid pterosaur [22], crocodilians, dinosaurs (abelisaurids, dromaeosaurids, titanosaurs, the iguanodontian *Rhabdodon*, the enantiornithine bird *Martinavis cruzyensis* and the giant ornithurine bird *Gargantuavis philoinos*) [23] [24 and references herein]. The fish record from the Cruzy localities consists of a lepisosteid indeterminate, and a mawsoniid represented by an angular, the first mawsoniid bone recognized in the Late Cretaceous of Europe [3]. The dinosaur assemblage indicates a Late Campanian–Early Maastrichtian age, an age confirmed by the dinosaur eggshells characteristic of the lower “Rogniacien” [25]. Based on sedimentological studies, the reconstructed environment was a braided river affected by flood episodes under a climate alternating dry and wet seasons [26].

#### Fox Amphoux—Bastide Neuve (Var) (FBN)

The composite faunal assemblage contains hybodont sharks, a lepisosteid and a mawsoniid fish (this study), the bothremydid *Foxemys mechinorum* [27], the eusuchian *Allodaposuchus* cf. *A*. *precedens* [28], an azhdarchid pterosaur [29] and dinosaurs (titanosaurids, dromaeosaurids [30], [31], abelisaurid [20], the iguanodontian *Rhabdodon*, ankylosaurs, enantiornithine birds [32] and the giant ornithurine bird *Gargantuavis philoinos* [33 and references herein] [34]. The vertebrate assemblage and an eggshell assemblage from the nearby locality of Métisson indicate a lower “Rognacien” age, corresponding probably to the Late Campanian [35]. Sedimentological and taphonomical data indicate a fluvial setting of moderate energy with broad floodplain palaeoenvironment.

#### Campagne-sur-Aude—Bellevue (Aude) (C3)

This locality has yielded hundreds of bones found during two decades of systematics excavations by the team of the Musée des Dinosaures from Espéraza. The fauna comprises lepisosteid and mawsoniid fishes (this work), turtles, crocodiles and dinosaurs including the titanosaur *Ampelosaurus atacis*, the iguanodontian *Rhabdodon*, nodosaurid, and avian and non-avian theropods [36], [37], [38]. The site is located in the upper part of the Maurine Red Marls Formation, regarded as the equivalent to the top of the «Bégudien» and base of the Rognacien facies in the Provence Bas-Languedoc area [39]. Recently, magnetostratigraphical data from the Haute Valley area indicated that the Bellevue bone bed is located in the ‘C32n.1n’ magnetostratigraphic zone, which corresponds to the Early Maastrichtian (ca 71.5 mya) [40]. Taphonomical and sedimentological features indicate that the environment of deposition was a meandering and braided fluvio-alluvial setting [41].

## Institutional abbreviations

**CD13-Pal**, Territorial collectivity of the Département des Bouches-du-Rhône, France; **Collection Méchin**, collection Patrick et Annie Méchin, Vitrolles, France; **Cruzy M**, Musée de Cruzy, France; **MDE**, Musée des Dinosaures d’Espéraza, France; **MHN.AIX.PV**, Muséum d’Histoire naturelle d’Aix-en-Provence, France; **MMS/VBN**, Musée du Moulin seigneurial of Velaux, France.

### Material

Basisphenoid, Bouc-Bel-Air–Sousquières (CD13-Pal.2019.6.1); parasphenoid, Velaux–La Bastide Neuve (MMS/VBN.09.001 G); fragment of an indeterminate tooth bearing bone, Campagne-sur-Aude–Bellevue (MDE C3-02-57); postparietal, Ollières–Autoroute Nord/Sud sites (MHN.AIX.PV.2019.13.1); supratemporals, Bouc-Bel-Air–Sousquières (CD13-Pal.2019.6.2 and -3); extrascapular, Ollières–Autoroute Nord/Sud sites (MHN.AIX.PV.2019.13.2); lachrymojugal, Bouc-Bel-Air–Sousquières (CD13-Pal.2019.6.4); squamosal, Bouc-Bel-Air–Sousquières (CD13-Pal.2019.6.5); preopercle, Bouc-Bel-Air–Sousquières (CD13-Pal.2019.6.10); opercle, Bouc-Bel-Air–Sousquières (CD13-Pal.2019.6.11); quadrate, Bouc-Bel-Air–Sousquières (CD13-Pal.2019.6.6); pterygoids, Bouc-Bel-Air–Sousquières (CD13-Pal.2019.6.7 and -8); fragment of pterygoid or prearticular or parasphenoid, Bouc-Bel-Air–Sousquières (CD13-Pal.2019.6.9); dentary, Cruzy–Massecaps (M2253); prearticular, Fox Amphoux–Bastide Neuve (collection Méchin 745); cleithra, Bouc-Bel-Air–Sousquières (CD13-Pal.2019.6.12 and -13).

All necessary permits were obtained for the described study, which complied with all relevant regulations (the Velaux Municipality, the environment department from CD 13, the territorial collectivity of the ‘Département des Bouches-du-Rhône’, the ‘Direction de l’Environnement, des Grands-Projets et de la Recherche’, the ‘Direction des Forêts et des Espaces Naturels’, the ‘Service Départemental d’Incendie et de Secours’).

## Systematic palaeontology

Actinistia Cope, 1871

Latimerioidei Schultze, 1993

Mawsoniidae Schultze, 1993

Genus *Axelrodichthys* Maisey, 1986

Type species: *Axelrodichthys araripensis* Maisey, 1986

*Axelrodichthys megadromos* Cavin, Valentin, Garcia, 2016

### Remark

In the present state, we are not able to distinguish distinct species within the bone sample collected in the various Campanian–Early Maastrichtian localities of Southern France. Consequently, we regard all this material as belonging to a single species, *Axelrodichthys megadromos*, keeping in mind that few bones are represented by several similar items. Further discoveries might show that several taxa of mawsoniids are actually present. In order avoid confusion in future studies, we provide an anatomical description that distinguished each locality.

### Ventabren—Aire de Repos

No new material from this locality is described herein. The specimens from the other localities are compared, as far as is it possible with the holotype from Ventabren. Pieces of this specimen are used for the reconstruction of the skull.

### Bouc-Bel-Air—Sousquières

This locality has yielded the most abundant material referred to *A*. *megadromos*.

**Fig 2:**
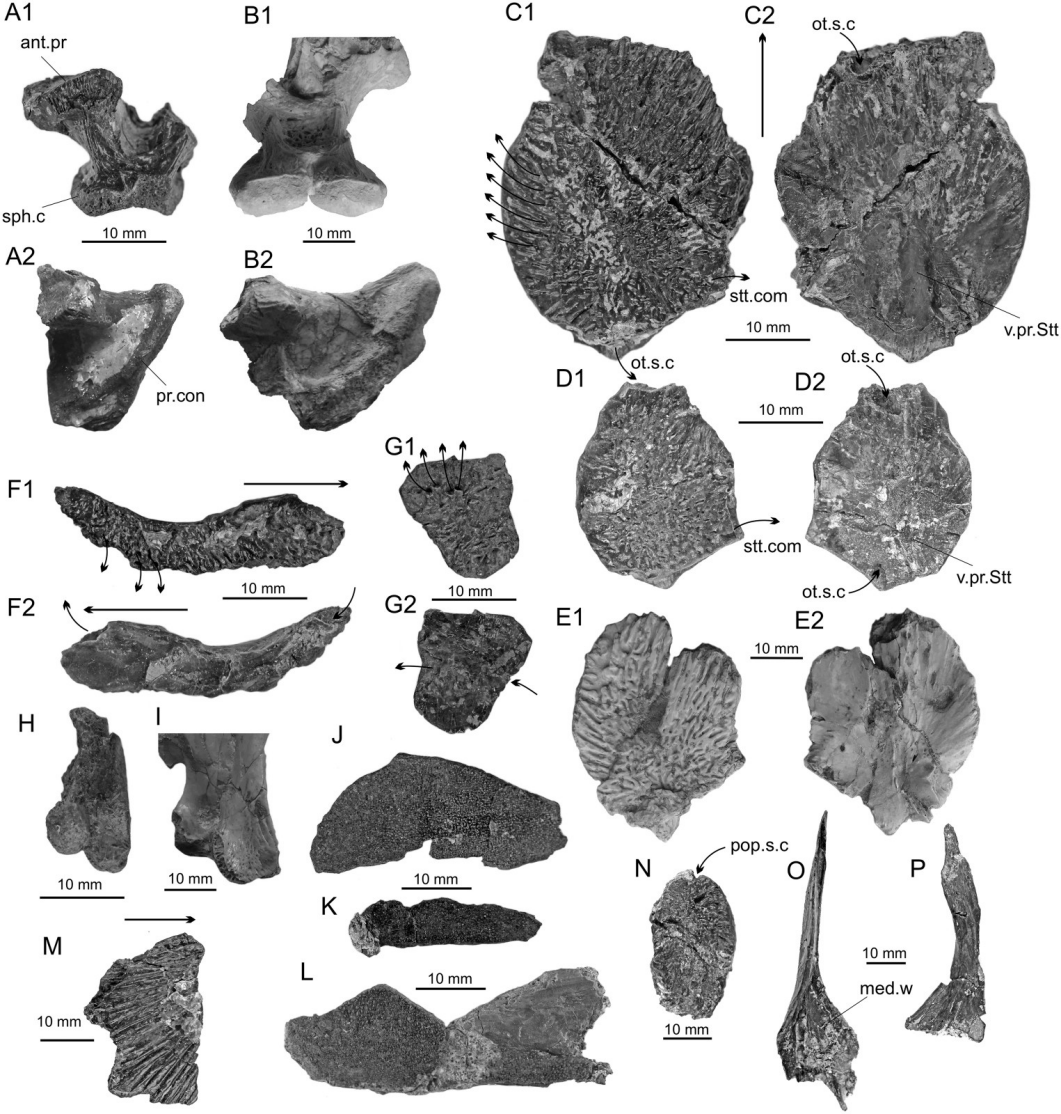
*Axelrodichthys megadromos*, Bouc-Bel-Air—Sousquières. **A**, basisphenoid from Bouc Bel Air (CD13-Pal.2019.6.1) in posterior (1) and left lateral (2) views; **B**, basisphenoid of *A*. *megadromos* from Ventabren (holotype, MDE F-61) in posterior (1) and right lateral (the photograph is inversed for easier comparison) (2) views; **C** and **D**, left supratemporals (CD13-Pal.2019.6.2 and -3) in external (1) and internal (2) views; **E**, right supratemporal (the photographs are inversed for easier comparison) of *A*. *megadromos* from Ventabren (holotype, MDE F-61) in external (1) and internal (2) views. This specimen was misidentified as a left postparietal in Cavin et al. (2016); **F**, right lachrymojugal (CD13-Pal.2019.6.4) in external (1) and internal (2) views; **G**, left (?) squamosal (CD13-Pal.2019.6.5) in external (1) and internal (2) views, **H**, left quadrate (CD13-Pal.2019.6.6) in anterior view; **I**, right quadrate (the photograph is inversed for easier comparison) in anterolateral view of *A*. *megadromos* from Ventabren (holotype, MDE F-61); **J-K**, pterygoids (CD13-Pal.2019.6.7, and -8) in medial view; **L**, pterygoid or prearticular or parasphenoid (CD13-Pal.2019.6.9) in occlusal view; **M**, fragment of opercle (CD13-Pal.2019.6.11); **N**, preopercle (CD13-Pal.2019.6.10) in lateral view; **O**, fragment of cleithrum (CD13-Pal.2019.6.12) in anterior view; **P**, fragment of cleithrum (CD13-Pal.2019.6.13) in lateral view. Abbreviations: ant.pr, antotic process; med.w, medial wing; ot.sc.c, otic sensory canal; pop.s.c, preopercular sensory canal; pr.con, processus connectens; sph.c, sphenoid condyle; stt.com, supratemporal commissure; v.pr.Stt, ventral or descending process of the supratemporal; Thin arrows indicate openings of the sensory canals and thick arrows indicate anterior.

Basisphenoid: A single, incomplete, basisphenoid is present (CD13-Pal.2019.6.1, Fig 2A). It is slightly smaller, but very similar in shape to the basisphenoid from the holotype of *A*. *megadromos* (Fig 2B), i.e. the antotic process protrudes laterally, the sphenoid condyles are close and moderately developed and they are squarish in dorsal view. In lateral view, the *processus connectens* is elongated and arched as in *A*. *megadromos*, but the sphenoid condyle is a little less protruding. This difference is possibly due to the imperfect state of preservation of the specimen from Bouc-Bel-Air.

Supratemporal: Two complete left supratemporals were found (CD13-Pal.2019.6.2 and -3, Fig 2C and 2D). One is about 1.5 times larger than the other. The ossifications are oval in shape with a regularly curved lateral margin. A well-marked angle separates the anterior margin from the anteromedial margin indicating that the median portion of the postparietal was longer than the lateral portion. The anterior margin is proportionally shorter on the small specimen. The suture between postparietal and supratemporal is interdigitate. The external (dorsal) surface of the bone is strongly ornamented with a reticulated pattern in the center of the ossification and irregular radiating rides at the periphery, which are more developed in the large specimen and especially marked along the lateral border. The internal (ventral) side is smooth, except a poorly developed descending process. The otic sensory canal enters the supratemporal close to the medial corner of the anterior margin of the bone and exits posteriorly at the posterior most tip of the bone, just above a little process. The otic sensory canal gives off lateral diverticula, which open and extend in deep grooves on the surface of the bone (arrows in Fig 2C). The supratemporal commissure is marked by an opening on the medial margin of the bone, close to its posterior corner. There are apparently no anterior extensions of the supratemporal commissure. The supratemporals from Bouc-Bel-Air are reminiscent of the supratemporal of all Cretaceous mawsoniid because of the strong ornamentation and the shallow descending process [1], [42], [43]. They are notably reminiscent of the supratemporal of a specimen from the Kem Kem beds, Morocco, referred to an indeterminate genus of mawsoniid sharing characters with *Axelrodichthys* [5]. Similarities between both specimens are the regularly curved lateral margin and the angle made by the suture with the posterior parietal. The well-preserved supratemporals from Bouc-Bel-Air allows re-identifying a bone belonging to the Ventabren type specimen originally identified as a left postparietal (Fig 2E). Because the margins of the bone from Ventabren are damaged, its original shape was supposedly more angled on the living fish, which led to conclude that it was a postparietal. Also, a small articular facet was regarded as a facet for intracranial joint less developed than in other mawsoniids [8]. This facet corresponds actually to the posterior small process present above the posterior opening of the otic sensory canal in the Bouc-Bel-Air supratemporals.

lachrymojugal: A comma-shaped bone is identified as a lachrymojugal (CD13-Pal.2019.6.4, Fig 2F). The external face is ornamented with very strong ridges and grooves. The dorsal margin, which marked the ventral border of the orbit, is regularly curved. One extremity is broader than the other. In most coelacanths, including the mawsoniids, the anterior extremity of the lachrymojugal is more expanded than the posterior one, which indicates that the bone described here is a right one. The infraorbital sensory canal is marked by two pores located at each extremity of the bone, better visible in internal view, and by at least three pores located within the strong ornamentation of the posterior half of the lateral face (arrows in Fig 2F). The lachrymojugal of *Mawsonia brasiliensis* is highly derived, with a very elongated straight posterior half and a curved anterior third, which marks the position of the orbit ([42] [43]). In *Axelrodichthys araripensis*, the dorsal margin of the lachrymojugal is curved along its whole length ([42] [1]) as in the bone from Bouc-Bel-Air, thus confirming the attribution of the French Late Cretaceous form to the genus *Axelrodichthys*.

Squamosal: An approximately trapezoidal bone, with a strong ornamentation, a series of pores aligned parallel to one of its margins and crossed by a sensory canal as indicate large openings visible in internal view, is tentatively identified as a squamosal (CD13-Pal.2019.6.5, Fig 2G).

Preopercle: An ovoid bone with a strong ornamentation and crossed along its long axis by a sensory canal is tentatively identified as a preopercle (CD13-Pal.2019.6.10, Fig 2N), because of is general shape reminiscent of the corresponding bone in *A*. *araripensis* ([1], [42]).

Other tick and ornamented dermal bone are present, but too badly preserved to be identified with accuracy.

Quadrate: A left articular head of a quadrate, showing the typical actinistian double and asymmetrical condyles for articulation with the lower jaw is present (CD13-Pal.2019.6.6, Fig 2H). It is similar in shape to the corresponding bone in the holotype of *A*. *maegadromos* (Fig 2I).

Pterygoid: Two plate-like fragments of bones covered with densely packed small bulbous teeth with radiating ridges are identified as pieces of pterygoids (CD13-Pal.2019.6.7 and -8, Fig 2J and 2K). This teeth morphology is typical of the mawsoniids, although it can also be found in other coelacanth genera. Another fragment, still attached to an indeterminate piece of bone, is covered with the same kind of teeth. It is tentatively identified as a pterygoid, although it could also be a fragment of prearticular or parasphenoid (CD13-Pal.2019.6.9, Fig 2L).

Opercle: A fragment of a thick bone with strong diverging ridges is identified as a piece of opercle (CD13-Pal.2019.6.11, Fig 2M), a bone which shows this typical radiating ornamentation in Brazilian ([42], [43], [44], [45]) and Moroccan [46], [47] mawsoniids.

Cleithrum: Two fragments corresponding to the dorsal half of cleithra with the beginning the medial expansion, typical of the lineage *Maswonia*–*Axelrodichthys* [1], are present (CD13-Pal.2019.6.12 and -13, Fig 2O and 2P).

### Ollières—Autoroute Nord/Sud sites

**Fig 3:**
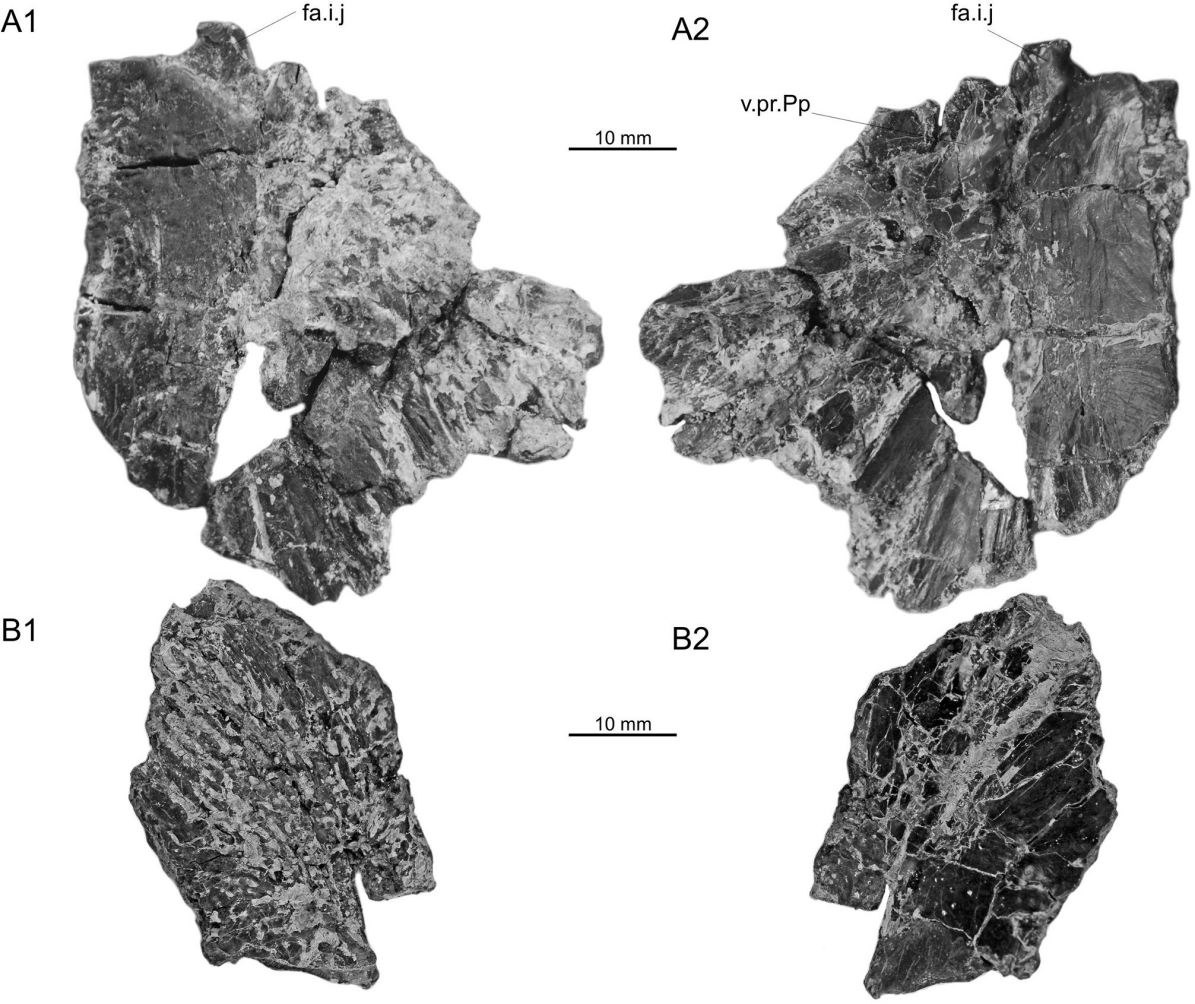
*Axelrodichthys megadromos*, Ollières—Autoroute Nord/Sud. A, right postparietal (MHN.AIX.PV.2019.13.1) in external (1) and internal (2) views; B, right extrascapular (MHN.AIX.PV.2019.13.2) in external (1) and internal (2) views. Abbreviations: fa.i.j, facet for the intracranial joint; v.pr.Pp, ventral or descending process of the postparietal.

Postparietal: A right postparietal is relatively poorly preserved but shows details of its general outline and of its anterior morphology (MHN.AIX.PV.2019.13.1, Fig 3A). It is the first known postparietal of *Axelrodichthys maegadromos* because the supposedly postparietal of the holotype specimen [8] is actually a supratemporal (see above). The external surface of the bone is worn and it is likely that the ornamentation has been partly erased after death. The anterior portion of the bone is smooth and grooves radiate on the posterior and lateral portions. The ossification was flattened during fossilization. As a consequence, the angle between the median horizontal portion and the lateral portion visible in anterior view, was more acute on the living fish than it is on the fossil (i.e. the lateral side was more inclined.) As a result, the postparietal as figured in Fig 3A is broader than it actually was. The descending lamina of the postparietal is damaged, but it was obviously weakly developed. The facet for the intracranial joint is well developed.

Extrascapular: a trapezoid bone with a strong ornamentation, found near the postparietal described above, is tentatively identified as an extrascapular (MHN.AIX.PV.2019.13.2, Fig 3B). The main feature supporting this identification is the presence of a groove along the posterior (?) margin, which correspond to the canal of the supratemporal commissure with a broken wall. Based on its general shape and proportions, it probably corresponds to the right lateral extrascapular. The reconstruction of the skull roof indicates that a small medial extrascapular was likely present, a character present in *Axelrodichthys* and absent in *Mawsonia* [42].

### Velaux—La Bastide Neuve

**Fig 4:**
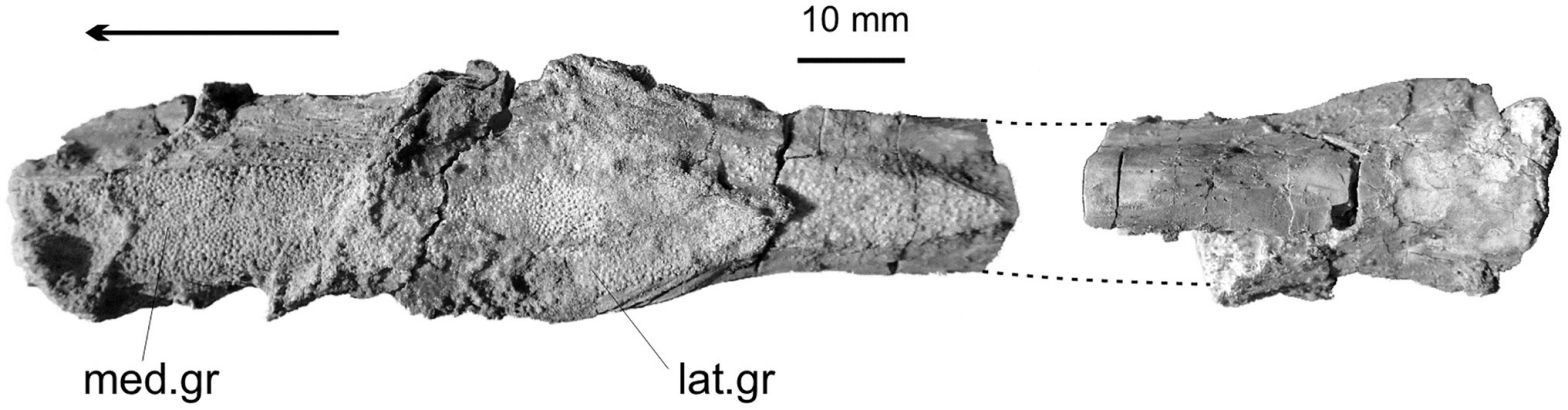
*Axelrodichthys megadromos*, Velaux—La Bastide Neuve. Parasphenoid (MMS/VBN.09.001 G) in ventral view. Thick arrow indicates anterior. Abbreviations: lat.gr, lateral groove; med.gr, medial groove.

**Fig 5:**
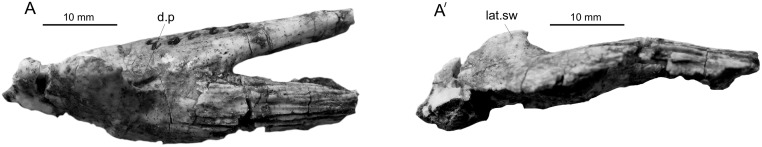
*Axelrodichthys megadromos*, Cruzy—Massecaps (M2253). Left dentary in lateral (A) and ventral (A’) views. Abbreviations: d.p, enlarged sensory pore; lat.sw, lateral swelling.

This locality has yielded a subcomplete parasphenoid (MMS/VBN.09.001 G, Fig 4). The posterior extremity of the bone and most of its anterior half are preserved, but no contact exists between both pieces. In ventral view, the posterior extremity broadens. Although poorly preserved, the section at this level of the bone forms a half circle. The posterior broken margin of the anterior portion is located at the level of the posterior extremity of the tooth patch. The tooth patch is tapering posteriorly. It broadens anteriorly and forms two lateral wings, which extend laterally the width of the ossification. At this level, two lateral longitudinal grooves separate a median ridge. At the anterior extremity of the preserved portion, the median ridge itself is dug by a medial depressed longitudinal area. In dorsal view, the anterior portion is only slightly curved in section but the curvature increases backwards. There is no shallow median ridge on the dorsal side as described by Wenz [47] in *‘Mawsonia’ lavocati*, but this difference may be caused by the different size of the specimens. The structure of the bone, in particular the contour of the posterior extremity of it toothed area, is similar in the corresponding bone in the holotype of *A*. *maegadromos* form Ventabren, in particular the heart-shaped posterior extremity of the patch, which is regarded as a diagnostic character of this species ([8]; Fig 5). We can add to the description of this bone in the holotype that the anterior portion of the median toothed ridge is dug by a central groove. The teeth are small and hemispherical, with a minute spiny apex on some of them. The radiating grooves on the teeth typical of mawsoniids are hardly visible, probably for preservational reason.

### Cruzy—Massecaps

The first recognised mawsoniid element from the Late Cretaceous of France, an isolated angular, was discovered in the locality of Massecaps in Cruzy [3]. The dentary described here comes from the same site (M2253, Fig 5). The bone is typical of dentaries of mawsoniids, i.e. it bears a hook-shaped process, a depression for the pseudomaxillary fold and a lateral swelling. The latter character is an apomorphy of the more derived mawsoniids (*Mawsonia*, *Axelrodichthys*, *Lualabaea*) [1]. Cupello et al. (2016) [45] showed that in *Mawsonia* the ventral extension of the dentary is broader and longer than the dorsal one, while in *Axelrodichthys* both extensions are almost equal in size, an arrangement also observed in M2253. The ventral body of the bone, which sutured with the splenial, is marked by deep grooves. An opening, corresponding to the enlarged sensory pore present in the dentary of some coelacanths, opens just posteriorly to the lateral swelling.

### Fox Amphoux—Bastide Neuve

**Fig 6:**
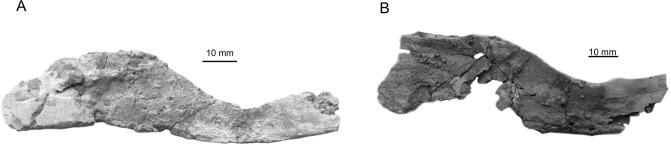
*Axelrodichthys megadromos*, Fox-Amphoux—Bastide-Neuve). **A**, right prearticular (collection Méchin 745) in medial view; **B**, right prearticular of *A*. *megadromos* from Ventabren (holotype, MDE F-61) in medial view.

A right prearticular (collection Méchin 745, Fig 6A) was found during recent excavations in the site of Bastide-Neuve. It is very similar to the less complete right prearticular of *A*. *megadromos* from Ventabren (Fig 6B). Both are thin bones with a subrectangular edentulous posterior lamina, which expands dorsally frontward, mirroring the general outline of the lateral angular (preserved in the holotype of *A*. *megadromos*). The deeper portion of the bone bears numerous tiny hemispherical teeth with the typical radiating ridges present in all mawsoniids.

### Campagne-sur-Aude—Bellevue

**Fig 7:**
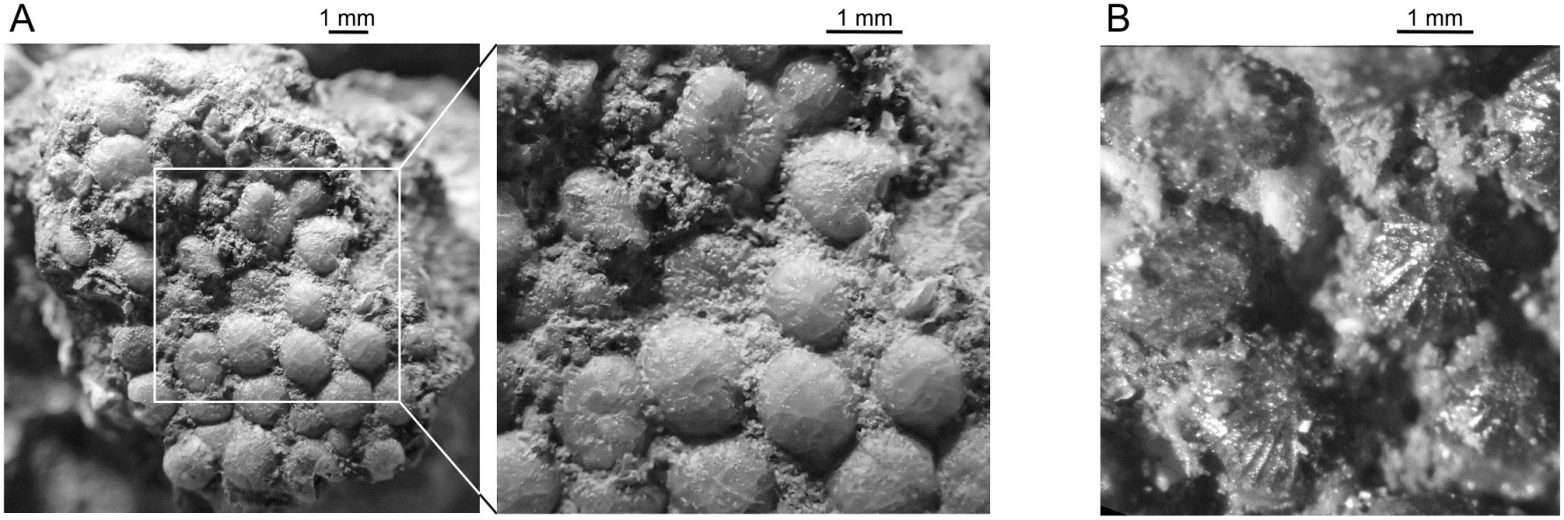
*Axelrodichthys megadromos*, Campagne-sur-Aude—Bellevue. **A**, fragment of a tooth patch, probably from a parasphenoid (MDE C3-02-57); **B**, Detail of teeth from a pterygoid from Bouc-Bel-Air (CD13-Pal.2019.6.9; Fig 2L) showing similar tooth morphology.

A small fragment, slightly less than 1 square centimetre, corresponds to a pavement of closely packed teeth (MDE C3-02-57, Fig 7A). The teeth are very similar in size and shape to the parasphenoid teeth from Velaux (compare Fig 4 with Fig 7A), or to teeth borne by an unidentified bone from Bouc-Bel-Air (Fig 7B is a detail of the specimen illustrated in Fig 2L), i.e. they are small, hemispherical and marked by fine striations that radiate from the apex. These features indicate that this piece likely belongs to a mawsoniid, likely a fragment of parasphenoid.

## Discussion

### Identification and affinities

Because all the mawsoniid material described in this study is incomplete or fragmentary, it is difficult to demonstrate that they all belong to a single species, i.e. *A*. *megadromos*. The fragmentary parasphenoid from Velaux, however shows the typical shape of the tooth patch of the parasphenoid of the type material of *A*. *megadromos*, a feature regarded as a diagnostic character [8]. The locality of Ventabren is situated at the base of the “Fuvélien” deposit (Early Campanian) while the locality of Velaux is situated in the “Bégudien” (late Campanian), indicating that approximatively 8 million years (Fig 1) separate both parasphenoids. Other bones know in duplicate, such as the basisphenoid and the supratemporal from the holotype of Ventabren and equivalent bones from Bouc-Bel-Air are also very similar, even though they do not show diagnostic characters of the species. The locality of Bouc-Bel-Air is situated in the same “Fuvélien” unit as Ventabren, making likely the occurrence in both sites of the same species. The issue of the presence of another species arises with the material from Cruzy, Fox-Amphoux and Campagne-sur-Aude because the former two sites are located in the “Rognacien” facies (probably late Campanian) and the latter site is located in the Maurine Red Marls (probably early Maastrichtian), i.e. these fishes are about 10 million years younger than the type specimen from Ventabren. However, duplicate bones from these younger localities, i.e. an angular from Cruzy, a prearticular from Fox-Amphoux and parasphenoid teeth from Campagne-sur-Aude show no characters differing enough from the type of *A*. *megadromos* for characterizing another species.

**Fig 8:**
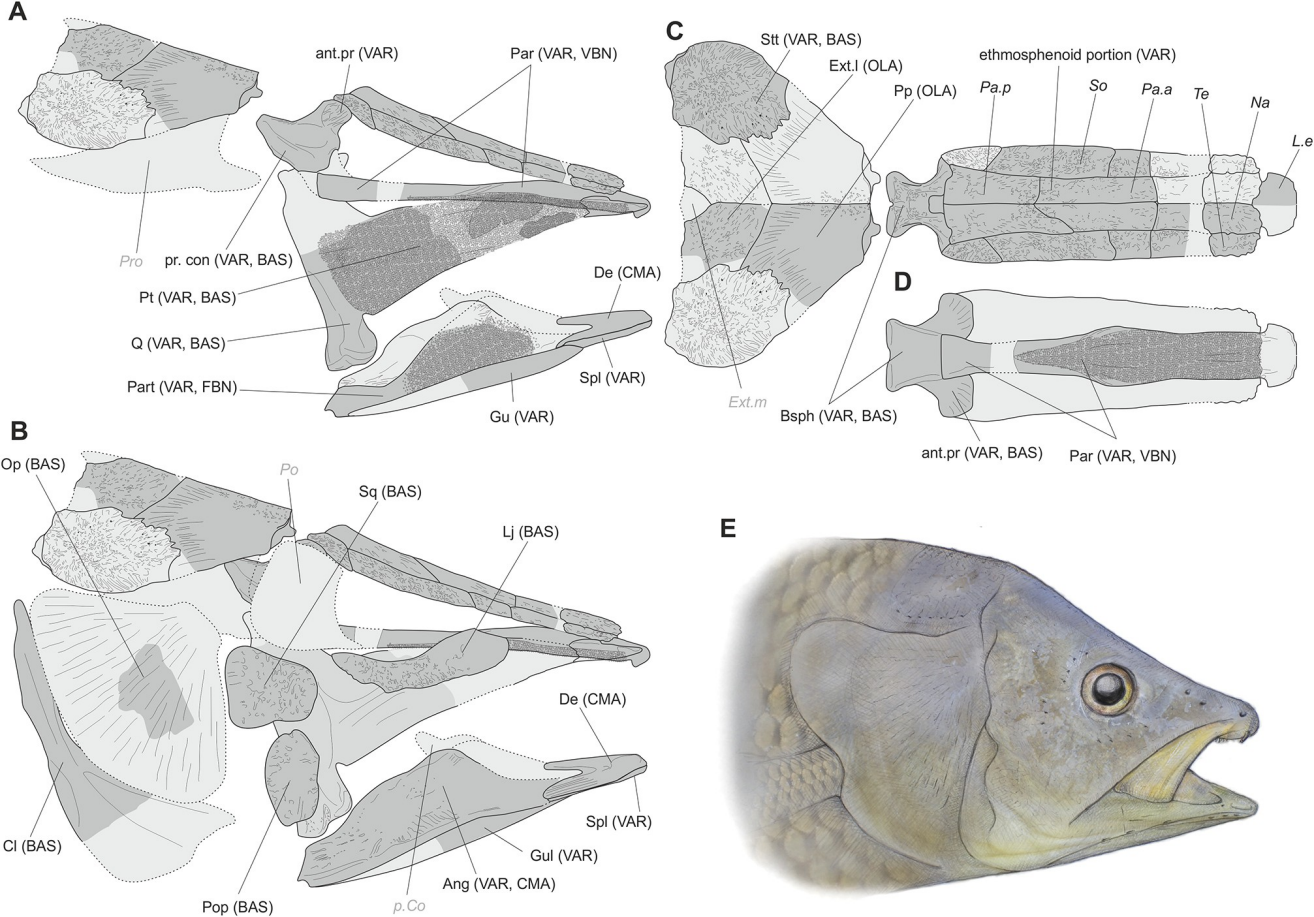
Reconstruction of the skull of *Axelrodichthys megadromos*. Dark grey bones correspond to preserved elements. For missing parts (light grey), the reconstruction is based on mirror effect of the preserved parts for paired bones and on bones preserved in *A*. *araripensis*, from the Albian Santana Formation, Brazil. Labels indicate names of bones and their corresponding locality in brackets. **A**, Braincase in right lateral view with the left suspensorium and lower jaw in medial view; **B**, skull with suspensorium, cheek and opercular elements in right lateral vie; **C**, skull roof in dorsal view; **D**, ethmosphenoid portion in ventral view. Abbreviations for bones: Ang, angular; ant.pr, antotic process; Bsph, basioccbasisphenoid; Cl, cleithrum; De, dentary; Ext.l, extrascapular lateral; Ext.m, extrascapular median; Gul, gular plate; L.e, lateral ethmoid; Lj, lachrymojugal; Na, nasal; Op, opercle; Pa.a, anterior parietal; Pa.p, posterior parietal; Par, parasphenoid; Part, prearticular; p.Co, principal coronoid; Po, postorbital; Pop, preopercle; Pp, postparietal; pr.con, processus connectens; Pro, prootic; Pt, pterygoid; Q, quadrate; So, supraorbital; Spl, splenial; Sq, suamosal; Stt, supratemporal; Te, tectal. Abbreviations for localities: BAS, Bouc-Bel-Air–Sousquières; CMA, Cruzy–Massecaps; FBN, Fox-Amphoux–Bastide-Neuve; OLA, Ollières–Autoroute North/South sites; VAR, Ventabren–Aire de repos; VBN, Velaux–La Bastide-Neuve.

Fig 8 shows a composite reconstruction of the skull of *A*. *megadromos*, with a tentative reconstruction of the head of the living animal. The reconstructed skull displays typical mawsoniid features, some of them present only in the *Mawsonia*-*Axelrodichthys* complex: the strongly ornamented bones of the skull roof and of the cheek, the shape of the postparietal and of the supratemporal and notably their shallow descending processes, the peculiar arrangement of the bones of the postparietal portion, with the extrascapulars included in the skull roof, the typical shape of the angular, the shape of the prearticular and pterygoid teeth, the lateral swelling of the dentary, and the medial expansion of the cleithrum. Other features, i.e. the probable presence of a medial extrascapular, the curved dorsal margin of the lachrymojugal, the ovoid shape of the preopercle, the posterior extensions of the dentary almost equal in size, and the general organization and proportions of the ethmosphenoid portion of the skull roof confirm the inclusion of this species in the genus *Axelrodichthys*.

**Fig 9:**
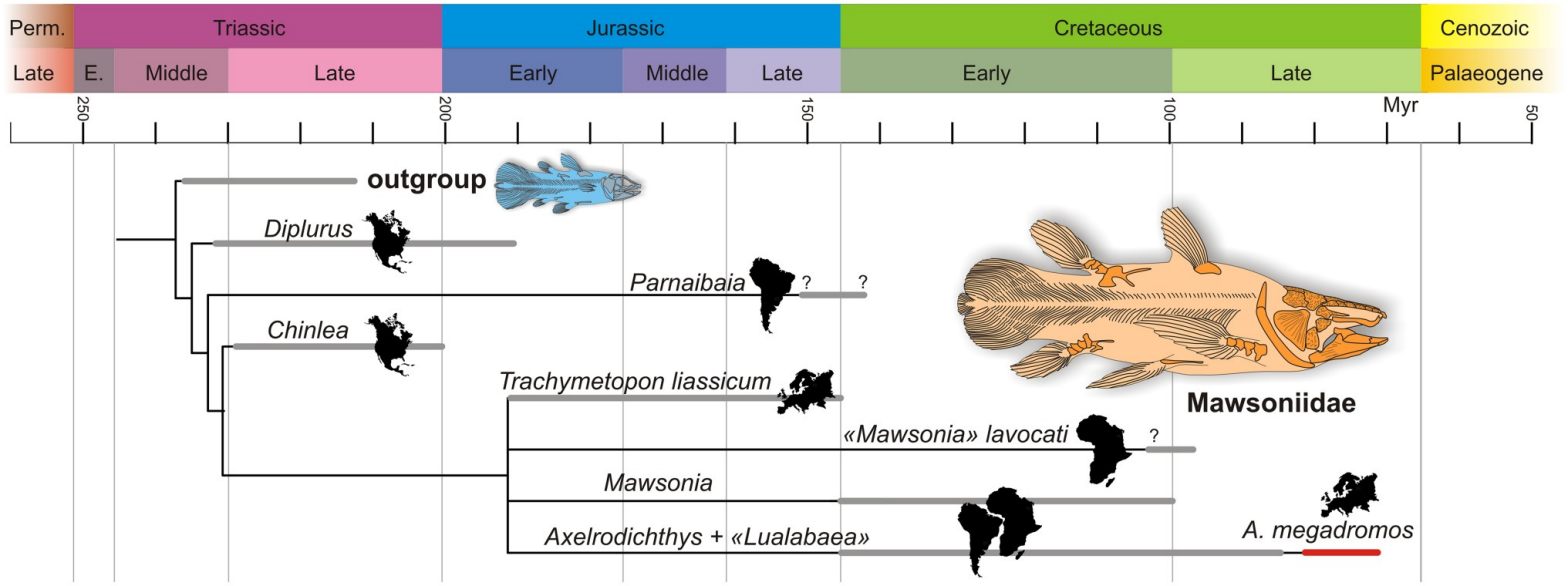
Simplified time-scaled phylogeny of the Mawsoniidae with their continental distribution (silhouettes). The trichotomy between three species of *Mawsonia* (*M*. *tegamensis*, *M*. *braziliensis* and *M*. *gigas*) and between two species of *Axelrodichthys* (*A*. *araripensis*, *A*. *megadromos*) and *Lualabea* are not figured.

In order to test the phylogenetic affinities of this species, we include *A*. *megadromos* in the recent phylogenetic cladistic analysis of Cavin et al. [7]. In the latter analysis, *A*. *megadromos* was resolved in a trichotomy with *A*. *araripensis* and *Lualabaea lerichei*, from the Early Cretaceous of Brazil and the Late Jurassic of the Democratic Republic of the Congo, respectively. The scoring of the character states of *A*. *maegadromos* was based on the holotype specimen only [8]. If we consider that all the Campanian–Maastrichtian mawsoniid material described here belong to this same species, we can now complete the scoring *A*. *megadromos* as follows: characters [state]: 1[2], 7[0], 8[0], 9[1], 10[1], 11[0], 17[2], 26[0], 27[0], 28[2], 29[0], 34[1], 48[0], 53[1] (for details of characters and character states definitions, see [7]). The new data matrix (S1 Data) was analysed using PAUP*4.0a167 (SWOFFORD, 2000) with a heuristic search using random addition sequence, replicated 10000 times, 10 trees held at each iteration, and tree bisection and reconnection branch swapping was carried out with *Latimeria* and *Macropoma* as outgroup. The exact topology and the amount of number of parsimonious trees are similar to the results obtained by [7] (Fig 9), and the indices are very close (18 MPT, length 100, CI = 0.660, RI = 0.663, RC = 0.438; previous metrics: 18 MPT, length 100, CI = 0.660, RI = 0.653, RC = 0.431). In consequence, the new material described herein brings new information for reconstructing the anatomy of this species, but it does not allow improving the understanding of its phylogenetic relationships. For a discussion of the distribution of the characters within the mawsoniid phylogeny, see [7].

### Ecological and biogeographical implications

The facies of all Campanian–Early Maastrichtian localities, in which *Axelrodichthys megadromos* was found indicate that this fish dwelled in lacustrine (the “Fuvélien” facies of Ventabren, Bouc-Bel-Air and Ollières), as well as in fluviatile and flood plain habitats (all other localities). None of these sites show marine influences, and this species should be regarded as a strictly freshwater fish. *A*. *megadromos* is so far the youngest known freshwater coelacanth worldwide (marine coelacanths are known in the Campanian–Maastrichtian of North America with the latimeriid *Megalocoelacanthus* [48], [49]).

Except *A*. *megadromos*, all other Cretaceous mawsoniid species occur in the Early Cretaceous of Western Gondwana, i.e. South America and Africa [7]. The youngest reasonably well-known species are ‘*Mawsonia’ lavocati* and *Mawsonia libyca* that were found in the Kem Kem beds of Morocco and in Bahariya in Egypt, respectively [7]. These two localities are generally regarded as Cenomanian in age [50], but we cannot ruled out a late Early Cretaceous age on the basis of the fish assemblage (Brito, personal communication, 2018). Most of the Western Gondwanan mawsoniid occurrences were found in freshwater environments, except *Axelrodichthys araripensis* and *Mawsonia brasiliensis*, which lived in the close marine and probably euryhaline environment of the Santana Formation in Brazil. The youngest Gondwanan mawsoniid remain is an isolated median extrascapular from the continental? Coniacian/Santonian Ankazomihaboka sandstones, Madagascar, referred to *Axelrodichthys* sp. by Gottfried et al. [2]. It is noteworthy that the last representatives of the mawsoniids have not been found in the geographical area were the family previously thrived, i.e. South America and Africa but in peripheral regions, i.e. Madagascar and Europe. It should be kept in mind that this observation rests in large part on the availability of continental Late Cretaceous outcrops, which are rather few in South America and Africa.

During the Late Cretaceous, on the European Archipelago some taxa were widespread across islands, e.g. the nodosaurid *Struthiosaurus*, the crocodilian *Allodaposuchus*, *Acynodon* and *Doratodon*, the turtles *Foxemys* among others, while other appear to be restricted to a single island, such as the solemydid turtles on the Ibero-Armorican landmass for instance [52]. These authors also noticed that the European vertebrates assembles is formed by a mix between an “old European core” to which was added immigrants from different bioprovinces, such as North America, Asia and Gondwana [20], [51], [52], [53]. The Ibero-Armorican landmass, in particular, shares taxa with Gondwana, such as characiform, mawsoniid and lepisosteiform fishes, neobatrachian frogs, bothremydid turtles and boiid snakes, ziphosuchian crocodyliforms, and abelisaurid dinosaurs [20], [54], [55], [56]. Dispersal events from Gondwana may have occurred during one, two or more continental connections between Africa and the European Archipelago during the Late Cretaceous. Csiki-Sava et al. [51] suggested that two dispersal events occurred for fishes, a pre-Campanian one involving lepisosteiforms, which are common in the Santonian site of Iharkút in Hungary [57], and one near the Campanian-Maastrichtian boundary involving characiforms and mawsoniids.

Le Loeuff et al. [58] were the first ones to recognise a turnover of European dinosaur assemblages during the Maastrichtian with an early Maastrichtian fauna dominated by titanosaurid sauropods and a late Maastrichtian fauna dominated by hadrosaurs. This observation was confirmed by several studies that focused on dinosaurs as a whole [40] and on sauropods [59]. Csiki-Sava et al. [51] confirmed, but nuanced this scenario, which applies mostly to the Ibero-Armorican Island. So far, the mawsoniid fossil record indicates that these fishes went extinct in Europe during this faunal turnover, with the youngest occurrence found in the early Maastrichtian site of Campagne-sur-Aude. If true, this scenario indicates that mawsoniids were extinct before the Cretaceous-Palaeogene mass extinction.

Eventually, it should be mention that the stratigraphical ranges of *A*. *megadromos* is relatively long, i.e. circa 10 million years, but it is still short compared to the stratigraphical range of *Mawsonia gigas*, which is circa 30 million years in South America [7]. These long ranges point to a slow morphological evolution and illustrates the status of ‘living fossils’ of the coelacanths taken as a whole, although with notable exceptions [60].

## Conclusion

Late Cretaceous European continental mawsoniids have been recognised only recently, about fifteen years ago. At that time, remains of these fishes appeared to be very rare, but examination of palaeontological collections from several localities reveals that they actually formed a non-negligible portion of the vertebrate assemblages. Fossils of mawsoniid coelacanths are more abundant and better preserved in the early Campanian “Fuvélien” facies than in the Late Campanian “Bégudien” and early Maastrichtian lower “Rognacien” facies. At the moment, we cannot decide if this trend: 1) corresponds to a real rarefaction of these fishes during this time interval; 2) is due to taphonomical or environmental biases linked to differences in the sedimentological and preservational features between facies or; 3) is caused by a sampling artefact.

The current data indicate the occurrence of mawsoniids only during a short time interval and in a restricted area of the Ibero-Armorican Island. But we hypothesize that new field discoveries and re-examination of palaeontological collections, especially from southern Pyreneans and Eastern Europe (Hungary and Romania) may enlarge the geographical range of the family. Similarly, it is likely that the stratigraphical range of mawsoniids will be extended towards the past, as indicate preliminary observation, but potentially towards the present as well. Mawsoniid fishes do not share the typical profile of the fish victims of the Cretaceous–Palaeogene mass extinction event [61], and it is not unlikely that representatives of this lineages crossed this boundary. So far, the fossil record of coelacanth is inexistent in the Cenozoic.

## Supporting information

S1 Data Character list and character-taxon matrix in nexus format. (NEX)
